# Efficacy, Safety, and Evaluation Criteria of mHealth Interventions for Depression: Systematic Review

**DOI:** 10.2196/46877

**Published:** 2023-09-27

**Authors:** Andrea Duarte-Díaz, Lilisbeth Perestelo-Pérez, Estel Gelabert, Noemí Robles, Antoni Pérez-Navarro, Josep Vidal-Alaball, Oriol Solà-Morales, Ariadna Sales Masnou, Carme Carrion

**Affiliations:** 1 Canary Islands Health Research Institute Foundation (FIISC) El Rosario Spain; 2 Network for Research on Chronicity, Primary Care and Health Promotion (RICAPPS) Madrid Spain; 3 The Spanish Network of Agencies for Health Technology Assessment and Services of the National Health System (RedETS) Madrid Spain; 4 Evaluation Unit (SESCS), Canary Islands Health Service (SCS) El Rosario Spain; 5 Department of Clinical and Health Psychology Universitat Autònoma de Barcelona Bellatera (Barcelona) Spain; 6 eHealth Center Universitat Oberta de Catalunya (UOC) Barcelona Spain; 7 Faculty of Computer Sciences, Multimedia and Telecommunication Universitat Oberta de Catalunya (UOC) Barcelona Spain; 8 eHealth Lab Research Group Universitat Oberta de Catalunya (UOC) Barcelona Spain; 9 Unitat de Suport a la Recerca de la Catalunya Central Fundació Institut Universitari per a la Recerca a l'Atenció Primària de Salut Jordi Gol i Gurina Sant Fruitós de Bages Spain; 10 Health Promotion in Rural Areas Research Group Gerencia Territorial de la Catalunya Central Institut Català de la Salut Sant Fruitós de Bages Spain; 11 Faculty of Medicine University of Vic-Central University of Catalonia Vic Spain; 12 Fundació HiTT Barcelona Spain; 13 Universitat Internacional de Catalunya (UIC) Barcelona Spain; 14 Office of Health Economics (OHE) London United Kingdom; 15 Estudis de Ciències de la Salut Universitat Oberta de Catalunya (UOC) Barcelona Spain; 16 eHealth Lab Research Group School of Health Sciences and eHealth Center Universitat Oberta de Catalunya (UOC) Barcelona Spain; 17 School of Medicine Universitat de Girona (UdG) Girona Spain

**Keywords:** mobile health, mHealth, apps, depression, systematic review, meta-analysis

## Abstract

**Background:**

Depression is a significant public health issue that can lead to considerable disability and reduced quality of life. With the rise of technology, mobile health (mHealth) interventions, particularly smartphone apps, are emerging as a promising approach for addressing depression. However, the lack of standardized evaluation tools and evidence-based principles for these interventions remains a concern.

**Objective:**

In this systematic review and meta-analysis, we aimed to evaluate the efficacy and safety of mHealth interventions for depression and identify the criteria and evaluation tools used for their assessment.

**Methods:**

A systematic review and meta-analysis of the literature was carried out following the recommendations of the PRISMA (Preferred Reporting Items for Systematic Reviews and Meta-Analyses) statement. Studies that recruited adult patients exhibiting elevated depressive symptoms or those diagnosed with depressive disorders and aimed to assess the effectiveness or safety of mHealth interventions were eligible for consideration. The primary outcome of interest was the reduction of depressive symptoms, and only randomized controlled trials (RCTs) were included in the analysis. The risk of bias in the original RCTs was assessed using version 2 of the Cochrane risk-of-bias tool for randomized trials.

**Results:**

A total of 29 RCTs were included in the analysis after a comprehensive search of electronic databases and manual searches. The efficacy of mHealth interventions in reducing depressive symptoms was assessed using a random effects meta-analysis. In total, 20 RCTs had an unclear risk of bias and 9 were assessed as having a high risk of bias. The most common element in mHealth interventions was psychoeducation, followed by goal setting and gamification strategies. The meta-analysis revealed a significant effect for mHealth interventions in reducing depressive symptoms compared with nonactive control (Hedges *g*=−0.62, 95% CI −0.87 to −0.37, *I*^2^=87%). Hybrid interventions that combined mHealth with face-to-face sessions were found to be the most effective. Three studies compared mHealth interventions with active controls and reported overall positive results. Safety analyses showed that most studies did not report any study-related adverse events.

**Conclusions:**

This review suggests that mHealth interventions can be effective in reducing depressive symptoms, with hybrid interventions achieving the best results. However, the high level of heterogeneity in the characteristics and components of mHealth interventions indicates the need for personalized approaches that consider individual differences, preferences, and needs. It is also important to prioritize evidence-based principles and standardized evaluation tools for mHealth interventions to ensure their efficacy and safety in the treatment of depression. Overall, the findings of this study support the use of mHealth interventions as a viable method for delivering mental health care.

**Trial Registration:**

PROSPERO CRD42022304684; https://www.crd.york.ac.uk/prospero/display_record.php?RecordID=304684

## Introduction

### Background

Depression is the most common mental health condition in the general population and is one of the leading causes of the global burden of disease and disability [1-3]. The worldwide incidence of depression increased by 49.86% between 1990 and 2017, from 172 million cases to 25.8 million [2]. Unipolar depression is predicted to be the leading cause of disability in high-income countries by 2030, surpassing other health conditions such as ischemic heart disease, dementia, alcohol use disorders, and diabetes [3].

Although there is strong clinical evidence that depression can be treated with a variety of pharmacological and psychological interventions [4], human resources for mental health are inadequate, especially in low- and middle-income countries [5-7], and a global shortage of over 15 million health workers is expected by 2030 [8]. Given the rapid advancement and adoption of technology, digital interventions—particularly mobile health (mHealth) interventions—have the potential to provide novel and viable methods of delivering population-scale mental health care [9,10].

The World Health Organization defines mHealth as “the term used for medical and public health practices supported by mobile devices, such as phones, patient monitoring devices, personal digital assistants, and other wireless devices” [11]. Smartphone apps can especially be powerful vectors for mHealth interventions because of their high connectivity, 24-hour availability, and ubiquitous nature [12]. Compared with most traditional treatment services, smartphone-based interventions offer several advantages, including high accessibility and scalability; relatively low costs; minimal contact; patient anonymity; flexibility of use; and the possibility of self-monitoring activity, symptoms, and progression in real time as well as providing motivational support and targeted care [10,13,14].

Self-management features are commonly found in mHealth interventions aimed at mental health problems, enabling clients to manage symptoms by monitoring their own symptoms and behavior [15]. In addition, mHealth apps for mood disorder management often provide stress-relieving games, meditation instructions, mood trackers, and psychoeducational materials. Despite the abundance of apps available in the commercial market for managing depressive symptoms, only a limited number incorporate a cognitive behavioral therapy (CBT) approach, despite CBT being widely recognized as a first-line psychological treatment [16].

Previous systematic reviews and meta-analyses have shown that smartphone-based interventions can have beneficial effects on clinical and nonclinical depressive symptoms in both general and clinical populations [9,17]. Moreover, digital interventions have been shown to be particularly effective, acceptable, feasible, and user friendly when embedded in a therapeutic context involving social interaction with mental health professionals to monitor progress and provide additional support [18]. A recent meta-review of meta-analyses concluded that apps for anxiety and depression produce definite clinical benefits, whether used for self-management or alongside professional guidance [12].

Several mHealth apps are currently available [19-21]. However, despite increased interest and use, no international standards or apps exist to evaluate mHealth apps in a simple and effective manner. Furthermore, although the number of mobile mental health apps is increasing owing to their convenience and high demand, many of these apps do not apply evidence-based principles or have not been tested for efficacy [16,22]. Therefore, selecting an app that is likely to be effective is problematic for users [9]. Health professionals and services are also increasingly using digital tools to facilitate disease management and need to be sure that the apps they recommend meet the minimum quality requirements [23]. Although several initiatives have been launched to define how mHealth apps should be assessed, these initiatives only address a part of the evaluation process and are mostly concerned with developing a methodology for evaluating all types of mHealth apps. As every health condition has specific needs, new tools and methodologies are required to evaluate apps targeting each condition.

### Objectives

This systematic review is part of the EvalDepApps research project [24], the primary objective of which is to develop and pilot an assessment tool for mobile apps aimed at treating and monitoring people with depressive symptoms. To that end, it is critical to comprehensively understand the effectiveness and safety of mHealth interventions based on available scientific evidence as well as the evaluation criteria used to measure these outcomes. Accordingly, the aims of this systematic review are (1) to assess how effective and safe mHealth interventions are in the treatment of depression and (2) to identify the criteria and evaluation tools used to assess these mHealth interventions.

## Methods

A systematic review and meta-analysis of the literature was performed following recommendations in the PRISMA (Preferred Reporting Items for Systematic Reviews and Meta-Analyses) statement [25] (Multimedia Appendix 1). The protocol for this systematic review and meta-analysis was prospectively registered on PROSPERO on February 19, 2022 (CRD42022304684).

### Search Strategy

A scoping search conducted to identify relevant search terms resulted in the following: “apps,” “mHealth,” “eHealth,” and “depression.” These were applied individually or combined according to Medical Subject Headings keyword terms in 3 electronic databases from inception to February 2022: MEDLINE, PsycINFO, and Embase. In addition, the reference lists of all eligible studies were screened to identify additional studies meeting the inclusion criteria.

### Inclusion and Exclusion Criteria

We considered studies recruiting adult patients with elevated depressive symptoms (ie, scoring above the cutoff criteria on a validated depression screening instrument) or diagnosed with depressive disorder (ie, diagnosed by a clinician or using any recognized diagnostic criteria). Studies recruiting children or adolescents aged ≤18 years were excluded. Studies assessing the effectiveness or safety of mHealth-based interventions for treating depression were included, whereas those using no mobile tools or relating to diagnosis or prevention were excluded. Studies referring to the management of other conditions, such as cancer, stroke, Alzheimer disease, epilepsy, social anxiety, alcoholism, or pain, were also excluded. Any comparator other than mHealth interventions was considered, including passive (eg, no intervention or waiting list) or active (eg, antidepressants or face-to-face psychotherapy) groups. The primary outcome was the reduction of depressive symptoms, and secondary outcomes included undesirable effects of the mHealth intervention and the criteria and evaluation tools used to assess the effectiveness and safety of mHealth interventions. Randomized controlled trials (RCTs) with at least 10 participants were included in the study design. Nonrandomized studies, uncontrolled studies, observational studies, conference abstracts, letters, commentaries, essays, book chapters, qualitative studies, study protocols, and reviews were excluded. We included studies published in English and Spanish, without imposing any restrictions on the publication year. Studies conducted in any country and clinical setting were considered.

### Risk-of-Bias Assessment

The risk of bias in the original RCTs was assessed using version 2 of the Cochrane risk-of-bias tool for randomized trials [26]. Quality assessment was performed by 2 independent reviewers, and any disagreements were resolved by consulting a third reviewer.

### Study Selection and Data Extraction

All citations extracted from electronic databases were imported into Rayyan, a web-based software program for systematic reviews, and duplicates were removed. Two members of the research team independently reviewed all titles and abstracts to preselect those systematic reviews meeting the inclusion criteria. The full texts of potentially relevant studies were screened for eligibility by 2 reviewers. Any disagreement was resolved by discussion and consensus, and a third reviewer was consulted, if required. Two reviewers then independently extracted data from each included RCT using a standardized data extraction form in Microsoft Excel using the following variables: (1) first author, (2) year of publication, (3) country, (4) number of participants, (5) study design, (6) study period, (7) study population, (8) intervention and control details, (9) outcome measures, and (10) main results. To gather information about the intervention details and elements included, we primarily relied on the descriptions of the interventions provided in the included studies. Furthermore, we also referred to other publications related to the same study, which offered a more comprehensive description of the intervention’s development process. In addition, when necessary, we consulted public descriptions available through websites or app stores.

### Data Synthesis and Analysis

Meta-analyses were performed using the inverse variance method [27] and were visually displayed using forest plots. A random effects model using the Sidik-Jonkman method as the tau estimator was applied [28]. Statistical heterogeneity between the different studies included in the meta-analyses was assessed using the Higgins *I*^2^ value [29]. For each meta-analysis, 2-tailed 95% prediction intervals were calculated. The following post hoc subgroup analyses were carried out: type of nonactive control, intervention length, depression severity at baseline, mHealth intervention framework, delivery mode, mood monitoring, goal setting, and gamification. Furthermore, the Galbraith plot was used to identify possible outliers, and a sensitivity analysis was performed using the leave-one-out function, which performs multiple meta-analyses excluding a single study at a time. We evaluated the publication bias using the Egger test [30], and the trim-and-fill method was used to correct for possible funnel plot asymmetry. All analyses were performed using Stata (version 17; StataCorp).

## Results

### Overview

**Figure 1:**
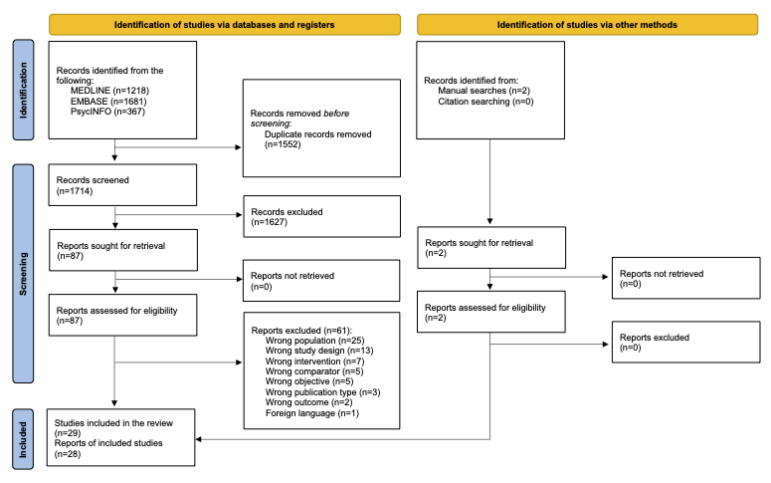
PRISMA (Preferred Reporting Items for Systematic Reviews and Meta-Analyses) flowchart of the selection process.

The initial search of the electronic databases yielded 3203 references. After removing duplicates, 1714 records were screened by title and abstract and 87 full-text articles were assessed for eligibility. Two additional records were identified through manual searches. Finally, 29 RCTs reported in 28 articles were included [31-58]. A flowchart of our selection process is shown in Figure 1.

### Characteristics of the Included Studies

The study included a total sample of 5594 participants, with an average age of 41.33 years and a majority of participants identifying as female (72%). Most studies were performed in Asia (8/29, 28%) [33-36,41,50,53,58], Europe (8/29, 28%) [37-40,44,51,52,56], and North America (8/29, 28%) [32,42,43,45,46,54,55,57], followed by Australia (3/29, 10%) [46,48,49] and South America (2/29, 7%) [31]. In most studies (18/29, 62%), participants had moderate depressive symptoms at baseline. The intervention period of the included studies ranged from 2 to 24 weeks, with an average of 8 weeks. A complete description of study characteristics is presented in Table 1.

**Table 1:** Characteristics of the included studies.

Study, year	Country	Sample size (intervention/control)^a^	Age (years), mean	Gender (women), %	Depression at baseline	Intervention	Control	Length (wk)
Araya et al [31], 2021	Brazil	880 (440/440)	56	86.5	Moderately severe depressive symptoms (PHQ-9^b^)	CONEMO	TAU^c^	6
Araya et al [31], 2021	Peru	432 (217/215)	59.7	81.5	Moderate depressive symptoms (PHQ-9)	CONEMO	TAU	6
Arean et al [32], 2016	United States	626 (211/209/206)	33.9	78.9	Moderate depressive symptoms (PHQ-9)	EVO Problem-Solving Therapy App (iPST)	Minimal intervention (health information)	4
Birney et al [43], 2016	United States	300 (150/150)	40.7	76.7	Moderate depressive symptoms (PHQ-9)	MoodHacker	Minimal intervention (health information)	6
Bruhns et al [52], 2021	Germany	423 (208/215)	23.0	78.5	Moderate depressive symptoms (PHQ-9)	Metacognitive Training (MCT) and more	Waiting list	4
Chan et al [53], 2021	Hong Kong	320 (167/153)	27.3	72.7	Severe depressive symptoms (CES-D^d^)	proACT-S	Waiting list	6
Dahne et al [54], 2019	United States	52 (24/19/9)	43.8	84.6	Moderately severe depressive symptoms (BDI-II^e^)	Moodivate MoodKit	TAU	8
Dahne et al [55], 2019	United States	42 (22/9/11)	36.1	66.7	Moderately severe depressive symptoms (BDI-II)	Aptívate iCouch CBT^f^	TAU	8
Ebert et al [56], 2018	Germany	204 (102/102)	44.2	80.4	Severe depressive symptoms (CES-D)	GET.ON Mood Enhancer	Waiting list	12
Graham et al [57], 2020	United States	146 (74/72)	42.3	82	Moderate depressive symptoms (PHQ-9)	IntelliCare	Waiting list	8
Guo et al [58], 2020	China	300 (150/150)	28.3	7.7	Moderate depressive symptoms (PHQ-9)	Run4Love (WeChat)	TAU	12
Ham et al [33], 2019	South Korea	80 (28/26/26)	44.2	85.7	Moderate depressive symptoms (BDI-II)	HARUToday (CBT) HARUToday (general)	Waiting list	10
Jannati et al [34], 2020	Iran	78 (39/39)	27	100	Moderate depressive symptoms (EPDS^g^)	Happy Mom	Waiting list	8
Kageyama et al [35], 2021	Japan	32 (16/16)	20.1	34.4	Moderate depressive symptoms (CES-D)	SPSRS	Waiting list	5
Lüdtke et al [37], 2018	Germany	90 (45/45)	42.9	78.4	Moderate depressive symptoms (PHQ-9)	Be Good to Yourself	Waiting list	4
Liu et al [36], 2022	China	83 (41/42)	23.1	55.4	Moderate depressive symptoms (PHQ-9)	XiaoNan (WeChat)	Bibliotherapy	16
Lukas et al [38], 2021	Germany	16 (5/11)	24.7	81	Moderately severe depressive symptoms (PHQ-9)	MT-Phoenix+face-to-face psychoeducation	Waiting list	2
Lukas et al [39], 2021	Germany	77 (40/37)	29.9	82	Moderate depressive symptoms (PHQ-9)	MT-Phoenix+face-to-face psychoeducation	Waiting list	2
Ly et al [40],2015	Sweden	93 (46/47)	30.6	69.9	Moderately severe depressive symptoms (PHQ-9)	Face-to-face behavioral activation (4 sessions) + smartphone app	Face-to-face behavioral activation (10 sessions)	10
Mantani et al [41], 2017	Japan	164 (81/83)	40.9	53.5	Moderate depressive symptoms (PHQ-9)	Kokoro-App+TAU	TAU	9
Pratap et al [42], 2018	United States	274 (112/83/79)	34.9	77.1	Moderate depressive symptoms (PHQ-9)	EVO iPST	Minimal intervention (health tips)	4
Raevuori et al [44], 2021	Finland	124 (63/61)	25.1	72.6	Moderate depressive symptoms (PHQ-9)	Meru Health Program + TAU	TAU	8
Roepke et al [45], 2015	United States	283 (93/97/93)	40.2	69.6	Severe depressive symptoms (CES-D)	SuperBetter CBT-PPT SuperBetter	Waiting list	4
Sawyer et al [46], 2019	Australia	133 (72/61)	31.1	100	Moderate depressive symptoms (EPDS)	eMums Plus	TAU	16
Stiles-Shields et al [47], 2019	United States	30 (10/10/10)	NR^h^	NR	Moderately severe depressive symptoms (PHQ-9)	Boost Me Thought Challenger	Waiting list	6
Tighe et al [48] (2017)	Australia	61 (31/30)	26.5	64	Moderately severe depressive symptoms (PHQ-9)	Ibobbly	Waiting list	6
Tønning et al [51], 2021	Denmark	120 (61/59)	43.9	31.6	Mild depressive symptoms (HDRS-17^i^)	MONSENSO	TAU	24
Watts et al [49], 2013	Australia	52 (22/30)	41	80	Moderate depressive symptoms (PHQ-9)	Mobile Therapy (Get Happy Program)	Computer therapy	8
Wong et al [50], 2021	China	79 (39/40)	32.9	84.8	Moderate depressive symptoms (PHQ-9)	Lifestyle Hub	Waiting list	9

^a^If there are 3 numbers, the first 2 numbers are intervention groups and the third one is the control group.

^b^PHQ-9: Patient Health Questionnaire-9.

^c^TAU: treatment as usual.

^d^CES-D: Center for Epidemiologic Studies Depression Scale.

^e^BDI-II: Beck Depression Inventory–II.

^f^CBT: cognitive behavioral therapy.

^g^EPDS: Edinburgh Postnatal Depression Scale.

^h^NR: not reported.

^i^HDRS-17: 17-item Hamilton Depression Rating Scale.

### Quality Assessment of the Included Studies

**Figure 2:**
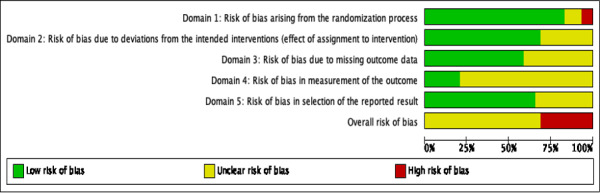
Risk-of-bias graph.

In total, 20 RCTs were identified as having an unclear risk of bias [31,32,34-36,40-42,44,46-53,56-58] and the overall risk of bias in the remaining 9 RCTs was assessed as high [33,37-39,43,45,49,54,55]. Depression symptoms were self-reported, and participants were mostly unblinded; therefore, the main sources of bias were the methods used to assess outcomes. A total of 12 RCTs [31,33,38,39,42,43,49-53,57,58] were judged to have an unclear risk of bias owing to missing outcome data. Most of the studies described treatment allocation as random, but 5 studies [38,39,43,54,55] did not provide enough details on the methods used to generate or conceal the sequence. Blinding is difficult with psychological mHealth interventions as participants are likely to be aware of what they are receiving. Nine studies reported in 8 references [31,33,42,45,47,49,54,55] that did not provide enough information about blinding or the method used to estimate the effect of assignment on the intervention were deemed to have unclear risks of bias due to deviations from the intended interventions. Most studies were reported in accordance with a prespecified plan and judged as having a low risk of bias in the selection of the reported result. A summary of the evaluation of risk of bias for each study is presented in Figure 2 in the form of a risk-of-bias graph with the opinions of review authors about each risk-of-bias item presented as percentages across all included studies.

### Elements Included in the mHealth Interventions

Psychoeducation about depression (17/31, 54%) was the most common element included in the mHealth interventions through videos, informative sheets, and even chatbots. The capability to set goals (13/31, 41%) and gamified strategies such as reward systems, challenges, and badges (12/31, 38%) were also prevalent. A substantial number of mHealth interventions (12/31, 38%) enabled users to receive professional support if required, and several interventions provided feedback on progress (10/31, 32%) and self-monitoring of mood (10/31, 32%). However, only 2 mHealth interventions (2/31, 6%) included components that encouraged user interaction through forums, chats, and other means. It is worth mentioning that most mHealth interventions (20/31, 64%) used regular reminders to increase adherence, retention, and ultimately effectiveness in reducing depressive symptoms. The specific characteristics and elements included in each mHealth intervention are presented in Multimedia Appendix 2 [31-58].

### Effectiveness of mHealth Interventions

#### mHealth Versus Nonactive Control

**Figure 3:**
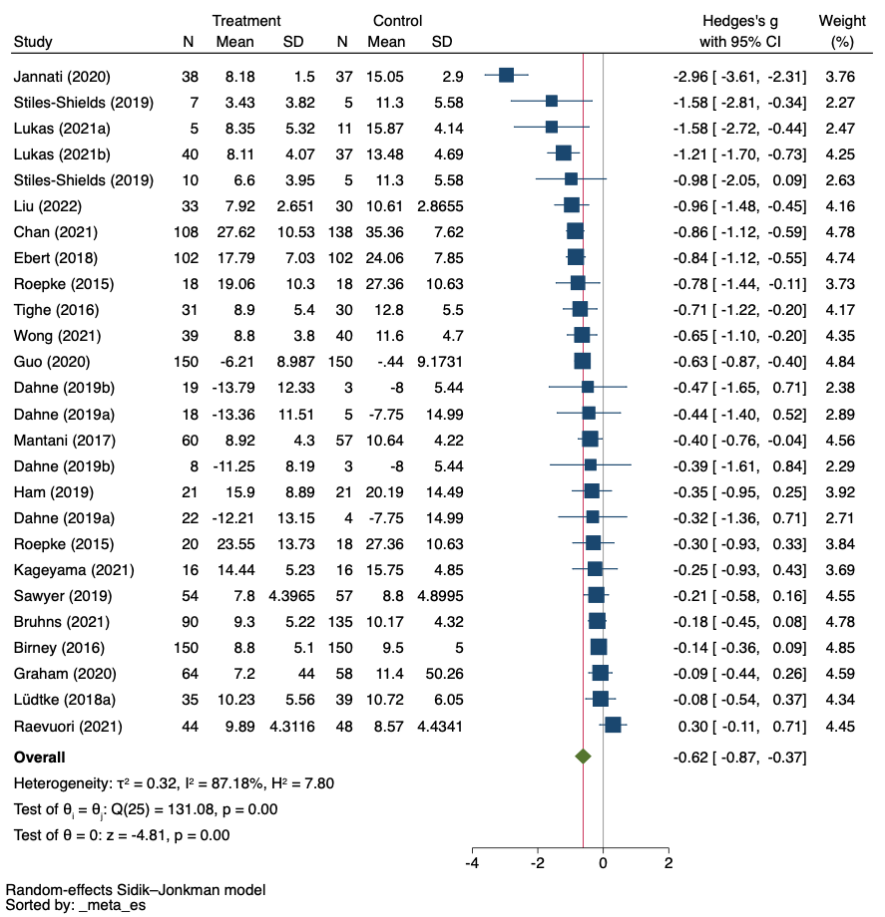
Random effects meta-analysis (mobile health vs nonactive control).

The meta-analysis of the effectiveness of mHealth for reducing depressive symptoms compared with nonactive controls included 26 effect sizes from 22 RCTs:16 compared mHealth with waiting list (n=1354), 6 with minimal intervention (n=145), and 4 with treatment as usual (TAU; n=620). The random effects meta-analysis showed a significant effect for mHealth (Hedges *g*=−0.62, 95% CI −0.87 to −0.37; *P*<.001; Figure 3). Heterogeneity across studies was high and statistically significant (*I*^2^=87%, Q=131.08, *P*=.001).

A subgroup analysis by type of nonactive control was not statistically significant (*P*=.12). However, the effect was higher in those studies comparing mHealth with minimal intervention or waiting list than in those comparing with TAU. A subgroup analysis by the severity of depressive symptoms at baseline was not statistically significant, although the effect was higher in people with moderately severe and severe depressive symptoms than in those with moderate depressive symptoms. Similarly, a univariate meta-regression using the baseline Patient Health Questionnaire-9 score as a moderator also displayed a trend toward significance, suggesting that people with higher depressive symptoms would benefit more from mHealth interventions (β=−.15, *P*=.08, k=14). Neither age nor gender was found to be significantly associated with higher effectiveness. In a subgroup analysis using the mHealth content framework, there were no statistically significant differences (*P*=.73), but CBT-based interventions were the most effective for reducing depressive symptoms, followed by acceptance-based interventions. Regarding the characteristics of mHealth interventions, only subgroup analysis by delivery mode was statistically significant (*P*=.03), with hybrid interventions—those combining mHealth with face-to-face sessions—showing the highest effect on reducing depressive symptoms. Univariate meta-regression by number of elements in the mHealth intervention was not statistically significant. More details on the subgroup analyses performed are presented in Table 2.

**Figure 4:**
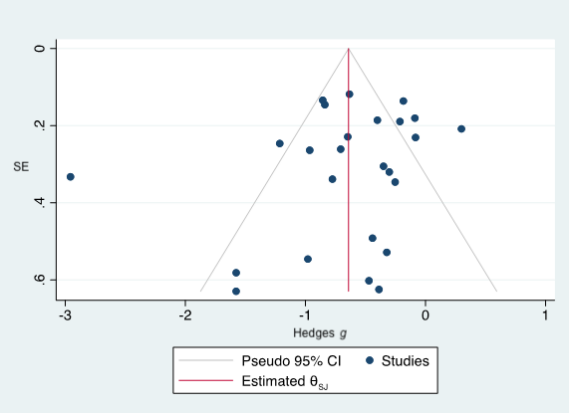
Funnel plot.

The funnel plot was symmetrical (Figure 4), trim-and-fill did not need to impute any additional study, and Egger tests showed no evidence of a small-study effect (*P*=.17).

The leave-one-out analyses suggest that the findings are robust, and neither the direction nor significance of the pooled effect changed after excluding any single study (Multimedia Appendix 3 [33-39,41,43-50,52-58]). However, the Galbraith plot identified 8 outliers that may have contributed to heterogeneity (Multimedia Appendix 4). The subsequent exclusion of outliers yielded a slightly lower pooled effect (Hedges *g*=−0.54, 95% CI −0.74 to −0.34, k=18), and heterogeneity was nonsignificant (*I*^2^=51%, Q=20.22, *P*=.26). None of the subgroup analyses or meta-regressions changed after the exclusion of outliers.

Four studies reported in 3 articles [31,32,43] compared mHealth interventions against nonactive controls but did not provide means and SDs and therefore were not included in the meta-analysis. In the 2 RCTs reported in the study by Araya et al [31], a digital intervention delivered over a 6-week period significantly improved depressive symptoms at 3 months when compared with usual care, but the magnitude of the effect was small in 1 trial, and the effects were not sustained at 6 months. According to Arean et al [32], mHealth apps designed to engage the cognitive correlates of depression have the greatest effect on reducing depressed mood in people with moderate levels of depression. In addition, Birney et al [43] found that the MoodHacker app produced significant effects on depression symptoms at the 6-week follow-up when compared with minimal intervention.

**Table 2:** Random effects models and subgroup analyses with depressive symptoms as the outcome.

Group		k	Hedges *g* (95% CI)	*I*^2^ (%)	Test for subgroup differences	
**Type of nonactive control**						Q=4.19; *P*=.12
	Minimal intervention	6	−0.41 (−0.77 to −0.09)	27.45		
	TAU^a^	4	−0.26 (−0.64 to 0.13)	81.0		
	Waiting list	16	−0.79 (−1.15 to −0.42)	89.17		
**Intervention length (weeks)**						Q=0.38; *P*=.54
	2-8	20	−0.66 (−0.98 to −0.33)	88.71		
	9-16	6	−0.53 (−0.76 to −0.30)	49.73		
**Depression severity at baseline**						Q=0.95; *P*=.62
	Moderate	14	−0.57 (−0.94 to −0.14)	93.81		
	Moderately severe	8	−0.77 (−1.19 to −0.35)	31.06		
	Severe	4	−0.77 (−1.02 to −0.51)	40.89		
**Measure**						Q=2.27; *P*=.52
	BDI-II^b^	5	−0.38 (−0.78 to 0.02)	0.00		
	CES-D^c^	5	−0.69 (−0.69 to −0.42)	48.83		
	EPDS^d^	2	−1.57 (−4.24 to 1.09)	98.02		
	PHQ-9^e^	14	−0.53 (−0.53 to −0.24)	85.09		
**Framework**						Q=2.02; *P*=.73
	Acceptance based	2	−0.73 (−1.14 to −0.33)	0.03		
	BA^f^	3	−0.59 (−1.25 to 0.06)	7.48		
	CBT^g^ only	10	−0.76 (−1.27 to −0.26)	94.56		
	CBT and others	8	−0.38 (−0.78 to 0.02)	85.07		
	Other	3	−0.70 (−1.40 to 0.01)	64.83		
**Delivery mode**						Q=6.87; *P*=.03
	App only	22	−0.57 (−0.85 to −0.30)	86.04		
	Hybrid intervention	2	−1.28 (−1.75 to −0.80)	4.47		
	Web and app	2	−0.48 (−1.15 to 0.19)	92.57		
**Psychoeducation**						Q=0.75; *P*=.39
	Yes	14	−0.69 (−1.12 to −0.27)	93.24		
	No	12	−0.48 (−0.71 to −0.25)	48.78		
**Mood monitoring**						Q=2.39; *P*=.12
	Yes	8	−0.37 (−0.59 to −0.15)	23.49		
	No	18	−0.69 (−1.04 to −0.35)	91.33		
**In-app feedback**						Q=0.97; *P*=.33
	Yes	8	−0.76 (−1.01 to −0.50)	70.56		
	No	18	−0.54 (−0.89 to −0.20)	87.62		
**Setting goals**						Q=0.13; *P*=.72
	Yes	10	−0.67 (−1.18 to −0.17)	95.37		
	No	16	−0.57 (−0.84 to −0.30)	68.12		
**Gamification**						Q=0.46; *P*=.50
	Yes	11	−0.52 (−0.79 to −0.25)	62.87		
	No	15	−0.68 (−1.07 to −0.29)	92.37		
**Professional support**						Q=0.02; *P*=.89
	Yes	9	−0.64 (−1.06 to −0.22)	87.81		
	No	17	−0.60 (−0.92 to −0.28)	86.61		

^a^TAU: treatment as usual.

^b^BDI-II: Beck Depression Inventory–II.

^c^CES-D: Center for Epidemiologic Studies Depression Scale.

^d^EPDS: Edinburgh Postnatal Depression Scale.

^e^PHQ-9: Patient Health Questionnaire-9.

^f^BA: behavioral activation.

^g^CBT: cognitive behavioral therapy.

#### mHealth Versus Active Control

Three studies compared mHealth interventions with active controls such as bibliotherapy [36], computerized CBT [49], and face-to-face behavioral activation [40]. Liu et al [36] found that a chatbot-delivered self-help depression intervention was superior to bibliotherapy in reducing depression. Watts et al [49] investigated whether a previously validated computerized program would be effective when delivered via a mobile phone app. Both the mobile and computer groups showed significant reductions in depressive symptoms at the 3-month follow-up. The authors concluded that the study provided preliminary evidence of clinically significant improvements in depressive symptoms when CBT is delivered via a mobile app. Ly et al [40] compared a hybrid treatment combining face-to-face behavioral activation and a smartphone app with a 10-session behavioral activation in people with major depression. Although both groups displayed significant improvements after 6 months of treatment, the hybrid intervention had reduced therapist time.

### Safety of mHealth Interventions

A total of 14 studies reported in 13 references [31,35,36,41-44,47,50,52,53,57,58] provided information on the safety of mHealth interventions. Most of these studies assessed safety by monitoring adverse events. Only Bruhns et al [52] and Mantani et al [41] included specific questionnaires: the Inventory for Assessing Negative Effects of Psychotherapy and Frequency, Intensity, and Burden of Side Effects Ratings, respectively. Overall, 9 of the 13 studies (69%) did not report any study-related adverse events. A complete description of the safety results in the included studies can be found in Multimedia Appendix 5 [31,35,36,41-44,47,50,52,53,57,58].

### Outcome Tools and Measures

The main end point outcome in the included studies was a reduction in depressive symptoms. However, several studies included secondary outcomes related to the effectiveness of mHealth interventions, such as quality of life, behavioral activation, and anxiety.

#### Quality of Life

A total of 11 studies out of 29 (37%) attempted to measure participants’ quality of life [31,33,37,40,44,50-53,58]. The World Health Organization Quality of Life—Abbreviated Version (4/11, 36%) was the most frequently used outcome tool, followed by the EQ-5D-3L (2/11, 18%), and the 36-item Short Form Survey (2/11, 18%). The Quality of Life Inventory, European Health Interview Survey–Quality of Life 8-Item Index, and 12-Item Short Form Survey were identified among the outcome tools in one study each. Overall, the results were inconsistent across studies, with 5 studies in 4 references reporting significant differences between groups in favor of the mHealth intervention for quality of life [31,50,51,58] and 6 studies reporting no significant differences between groups [33,37,40,44,52,53].

#### Anxiety

Nine of 29 studies (31%) also included anxiety as an outcome measure [33,35,36,40,44,45,50,53,56]. The most frequently used tool was the Generalized Anxiety Disorder-7 (5/9, 56%), followed by the anxiety subscale of the Hospital Anxiety and Depression Scale (2/9, 22%). The State-Trait Anxiety Inventory (1/9, 11%) and Beck Anxiety Inventory (1/9, 11%) were used in one study each. Six studies found that mHealth interventions significantly reduced anxiety symptoms compared with the waiting list [33,35,40,44,45,50,53,56] or bibliotherapy [36].

#### Perceived Stress

Perceived stress was assessed in 7 studies (24%) [35,44,46,48,49,51,58]. Three of these used the Perceived Stress Scale, another 3 used 6- or 10-item versions of the Kessler Screening Scale for Psychological Distress, and 1 assessed parenting stress through the Parenting Stress Index. Results were inconsistent, with 4 observing significant stress reductions with the mHealth interventions compared with the control group [44,48,49,58], and 3 indicating no significant effects [35,46,51].

#### Disability

According to the World Health Organization, depression is a leading cause of disability worldwide and a major contributor to the overall global burden of disease. Disability was measured in 6 (20%) out of 29 studies [31,32,42,49,50]: the Sheehan Disability Scale was used in 3 studies (N=6, 50%), the World Health Organization Disability Assessment Schedule II was used in 2 (N=6, 33%), and 1 used the Disability Symptom Severity (N=6, 16%). Three studies in 2 references found significant effects [31,51], whereas 3 others did not [32,42,49]. Therefore, mHealth interventions have not been conclusively proven to reduce depression-related disability.

#### Behavioral Activation

As a person with depression may withdraw from their surroundings and disengage from their routines, thus reducing opportunities for positive reinforcement, many depression interventions have included behavioral activation as a goal. Four of the 29 studies (13%) [31,51,56] assessed behavioral activation using the Short Form of the Behavioral Activation for Depression Scale, and 3 of these found statistically significant differences between mHealth interventions and control groups.

#### Insomnia

In 4 of 29 studies (13%), insomnia was measured using the Insomnia Severity Index [44,50,53,56]. Significant between-group differences favoring the mHealth intervention were found in 3 of these studies (low to large effect sizes compared with the waiting list) [50,53,56]. In contrast, Raevuori et al [44] found no significant differences in sleep disturbance between a group receiving mHealth plus usual care and a control group receiving usual care alone.

#### Self-Efficacy

Three studies (N=29, 10%) assessed the effectiveness of mHealth interventions on self-efficacy [45,46,58]. Measures used included the General Self-Efficacy Scale, Self-Efficacy Scale, and Parental Sense of Competence Scale. Both studies using general self-efficacy measures found significant results favoring mHealth interventions [45,58], but no effect on parental competence was found [46].

#### Self-Esteem

The Rosenberg Self-esteem Scale was used in 2 studies (N=29, 6.9%) that compared mHealth interventions with waiting list controls. Although Bruhns et al [52] found a medium to large effect size favoring smartphone self-help apps, Lüdtke et al [37] found no statistically significant differences between groups.

#### Other Outcome Tools and Measures

Each of the following outcome measures was assessed and described in a single study (1/29, 3%): knowledge of depression (self-developed scale) [43]; problem-solving (Social Problem-Solving Inventory–Revised) [56]; mastery (Pearlin Mastery Scale) [56]; negative thinking (Automatic Thoughts Questionnaire–Revised) [43]; coping (Simplified Ways of Coping Questionnaire) [58]; physical activity (Global Physical Activity Questionnaire) [58]; dysfunctional attitudes (Dysfunctional Attitudes Scale) [33]; affect (The Positive and Negative Affect Schedule) [36]; well-being (World Health Organization Well-being Index) [39]; psychological inflexibility and experiential avoidance (Acceptance and Action Questionnaire) [40]; resilience (Resilience Scale) [44]; satisfaction with life (Satisfaction with Life Scale) [45]; impulsivity (The Barratt Impulsivity Scale) [48]; suicidality (Depressive Symptom Inventory–Suicidality Subscale) [48]; psychological functioning (Functional Assessment Short Test) [51]; empowerment (Roger’s Empowerment Scale) [51]; and worry (Penn State Worry Questionnaire) [51]. The results for these outcomes can be found in Table S1 in Multimedia Appendix 6 [31-33,35-40,42-46,48-53,56,58].

### Output Tools and Measures

Although the main aim of the selected studies was to measure the effectiveness of mHealth interventions in reducing depressive symptoms, most also measured other outputs that could be relevant in determining primary outcome measures, such as adherence and app use, acceptability, and usability. The results for these outputs can be found in Table S2 in Multimedia Appendix 6.

## Discussion

### Principal Findings

Our review assessed 29 studies reported in 28 articles involving a substantial number of adult patients with elevated depressive symptoms or diagnosed depression. The meta-analysis of 26 studies comparing the effectiveness of mHealth interventions with the waiting list, minimal intervention, and TAU found moderate positive effects (Hedges *g*=−0.62) for mHealth, despite high levels of heterogeneity. These results align with those of 2 earlier meta-analyses comparing the efficacy of mHealth interventions and nonactive controls on reducing depressive symptoms, which showed effects of Hedges *g*=−0.56 and Hedges *g*=−0.51 [9,59]. However, these are higher than findings from other studies that included patients with any mental health issue (Hedges *g*=−0.33) [60] and compared mHealth with active treatments (Hedges *g*=−0.22) [9]. Owing to high heterogeneity and the small number of studies, conducting a meta-analysis to compare mHealth with other active interventions was not feasible.

The dynamic between health care professionals and patients is undergoing transformation owing to the influence of numerous technological, social, and environmental factors, leading to an evolving and changing relationship [61]. As mental health care delivery evolves toward a hybrid model incorporating both in-person and online interventions for diagnosis, therapy, and monitoring, the use of mobile devices becomes increasingly crucial, serving as an integral component in the assessment and intervention of mental health problems [62,63]. Although the number of studies assessing this type of intervention is small, the available evidence suggests that a combination of these 2 modalities can lead to better outcomes for individuals with depression. A potential explanation for the superior efficacy of hybrid therapy is the synergistic combination of app-based and face-to-face interventions. Although app-based interventions provide access to therapeutic content and activities at any time, face-to-face therapy has the advantages of personal interaction, direct guidance, and a supportive environment. An integration of these modalities provides a comprehensive treatment experience for individuals with depression, which may improve the therapeutic process and lead to better outcomes. Furthermore, the complementary nature of the 2 interventions may enhance the reinforcement of skills and strategies learned in face-to-face therapy as well as provide ongoing support and accountability, thereby potentially improving long-term symptom management. As highlighted by Ly et al [40], this could be explained by the dose-response effect, wherein lower doses of psychotherapy have been associated with poorer outcomes [64]. Moreover, hybrid therapy has the potential to be more cost-effective than traditional face-to-face treatments by combining in-person and on the web or app-based sessions, reducing medical costs per patient and increasing the capacity of therapists to treat more individuals with depression, thereby expanding access to treatment. Despite the crucial importance of implementation costs and cost-effectiveness in determining the feasibility and scalability of digital and hybrid interventions, there is a lack of sufficient evidence to date, and additional research is required to inform public and private reimbursement systems and enable investment in digital interventions.

In terms of app design, our findings suggest that incorporating CBT and acceptance frameworks can lead to a greater reduction in depressive symptoms. However, subgroup analyses by theoretical framework did not show statistically significant differences. This is consistent with existing evidence on the effectiveness of psychological interventions. Although CBT is one of the main nonpharmacological treatment options for depressive disorders, a recent network meta-analysis covering efficacy, acceptability, and long-term outcomes found little difference in results from various types of psychotherapy and concluded that most are effective and acceptable for treating adult depression [65]. Clearly, it is essential to design mHealth interventions based on evidence-based frameworks to guarantee their foundation in robust and reliable scientific evidence, and studies have highlighted the need for future research to better characterize the app features that maximize therapeutic effects [66]. However, we found that none of the individual elements in the apps (ie, psychoeducation, mood monitoring, in-app feedback, goal setting, gamification, and professional support) was significantly associated with a greater reduction in depressive symptoms. Moreover, mHealth interventions with a larger number of components are not always more successful: in some cases, simpler interventions that focus on a limited number of well-implemented and user-centered elements can be more effective. It is thus necessary to move beyond “one-size-fits-all” approaches in the design and delivery of mHealth interventions and prioritize tailored approaches that consider individual differences, needs, and preferences [67,68].

With the goal of identifying which sociodemographic and clinical characteristics of patients were associated with greater app effectiveness, we performed subgroup analyses and meta-regressions for gender, age, and baseline depression symptom severity variables. Our results show that mHealth interventions have been effective across demographic factors but may be more effective for individuals with moderate to severe depressive symptoms than for those with lower symptom levels. This is consistent with the results of a previous systematic review [59]. Furthermore, it is in line with the findings of other studies that have concluded that individuals with severe burden benefit equally or to a greater extent from low-intensity internet- and mobile-based interventions [69-71]. There are several potential explanations for these findings. One possibility is that patients with more symptoms have a greater capacity for definable and noticeable improvement. In addition, people with moderate to severe depressive symptoms may be more motivated to engage in psychological interventions and more likely to adhere to a treatment plan.

The disparity between RCT data and individual patient characteristics encountered in real-world health care settings is a widely acknowledged challenge in daily clinical practice [72]. To ensure the ultimate success of the mental health technology revolution, it is imperative to bolster the path toward the evaluation of implementation, bridging the gap between research findings and the unique features of each patient [73]. Although RCTs have demonstrated the effectiveness of digital interventions for addressing common mental health issues, it is crucial to shift our focus beyond these controlled settings. Unfortunately, there is a scarcity of reported data regarding the implementation of these interventions in the real-world context. The limited available data suggest that uptake and engagement vary widely among the handful of implemented digital self-help apps and programs that have reported this and that use may vary from that reported in trials [74]. It is essential to assess how these mHealth tools are used in real-life scenarios and to determine the extent to which their effectiveness endures beyond the controlled environment of research studies. This exploration beyond RCTs will provide valuable insights into their practical impact, accessibility, and overall contribution to enhancing the mental health of the population.

The increase in the use of mHealth apps has outstripped the development of international standards or practical evaluation tools to assess their effectiveness in a comprehensive and efficient manner. Despite a plethora of mHealth interventions, few have undergone rigorous scientific evaluation. In addition, most mHealth apps that have encountered any evaluation have only undergone a single study, typically with a small sample size. Only a minority of the mHealth interventions identified in our review have been subjected to evaluation in more than one study. Our results do indicate consistency in the assessment of depressive symptoms, as most studies use established and validated measurement tools, such as the Beck Depression Inventory–II, Patient Health Questionnaire-9, and Hospital Anxiety and Depression Scale. However, given the high heterogeneity of the identified measures, there appears to be a lack of consensus on how to assess other important outputs that are crucial in determining primary outcome measures, such as adherence, acceptability, usability, and app use. Furthermore, the absence of adequate regulatory bodies to oversee and regulate app development and availability has made accessing trustworthy and validated mHealth interventions a challenging process [21]. Accordingly, there remains a requirement for the development of new tools and methodologies that facilitate the assessment of various aspects of mHealth interventions intended to manage specific conditions. The results of this SR enable us to understand the effectiveness and safety of apps targeting depression that have been evaluated in RCTs, as well as the evaluation criteria used, and will serve as a starting point for the design of an evaluation tool within the context of the EvalDepApps research project [24].

### Strengths and Limitations of This Study

Our study has several key strengths, including a rigorous and systematic search and selection process that ensured comprehensive coverage of the available evidence. Furthermore, the use of validated quality assessment tools facilitated a robust evaluation of the risk of bias in the included studies. Clear and transparent reporting of methods and results enhances the reproducibility of the findings and strengthens the validity and reliability of the results. However, there are also several limitations that should be considered when interpreting our results. Our search for studies was limited to those published in English or Spanish and did not incorporate gray literature, which may have excluded some relevant studies. It should also be noted that most of the reviewed studies were conducted in Western high-income countries; thus, it is unclear whether these results can be generalized to low- and middle-income countries. Our analyses revealed moderate heterogeneity that could not be fully accounted for through subgroup analyses. This heterogeneity may be due to differences in populations, interventions (including the framework, elements included, and definitions of these elements), and comparators across the trials. For example, we compared mHealth interventions with a variety of control conditions, including waiting list, minimal intervention, and TAU. Although we found no significant differences between these control conditions, the variability among them may have contributed to the overall heterogeneity. Another noteworthy limitation of our review was the exclusion of studies that did not present results from RCTs. Although observational studies and nonrandomized trials could potentially offer valuable insights into the practical use and effectiveness of mHealth in the real-world context, we decided to exclude them because of the higher susceptibility of these trial designs to various biases, which may compromise the reliability of the findings. Finally, there are important limitations associated with the small sample sizes and moderate to high risk of bias present in most of the studies reviewed.

### Conclusions

This study suggests that mHealth interventions directed toward adults experiencing elevated symptoms of depression result in moderate decreases in these symptoms, regardless of age and gender, with hybrid interventions achieving the best results. However, it should be noted that most of the studies in this review had small sample sizes and were associated with a moderate to high risk of bias. In addition, a high level of heterogeneity was observed in the characteristics and components of the mHealth interventions, with no singular element found to be associated with improved outcomes. Hence, it is imperative to move beyond generic solutions when designing and delivering mHealth interventions and prioritize individualized approaches that take into consideration individual differences, needs, and preferences.

## Appendix

Multimedia Appendix 1 PRISMA (Preferred Reporting Items for Systematic Reviews and Meta-Analyses) checklist.

Multimedia Appendix 2 Characteristics and elements included in the mobile health interventions.

Multimedia Appendix 3 Sensitivity analysis.

Multimedia Appendix 4 Galbraith plot.

Multimedia Appendix 5 Safety of the mobile health interventions.

Multimedia Appendix 6 Secondary outcome results from the selected studies: output tools and results from the selected studies.

## Abbreviations

CBT: cognitive behavioral therapy
mHealth: mobile health
PRISMA: Preferred Reporting Items for Systematic Reviews and Meta-Analyses
RCT: randomized controlled trial
TAU: treatment as usual
