# Supplementary material for: Efficacy, Safety, and Evaluation Criteria of mHealth Interventions for Depression: Systematic Review
Source: JMIR Ment Health. 2023 Sep 27;10:e46877. doi: 10.2196/46877 (PMC10568392; doi:10.2196/46877)
Supplement: Multimedia Appendix 2 [file mental_v10i1e46877_app2.docx]

Multimedia Appendix 2. Characteristics and elements included in the mHealth interventions

| **mHealth intervention** | **Study** | **Framework** | **Elements included** | | | | | | | |
| --- | --- | --- | --- | --- | --- | --- | --- | --- | --- | --- |
|  |  |  | **Psychoeducation** | **Self-monitoring** | **Setting goals** | **Feedback** | **Gamification** | **Peers network** | **Professional support** | **Reminders** |
| CONEMO | Araya (2021) | BA | + | - | + | + | - | - | + | - |
| EVO | Arean (2016)  Pratap (2018) | Other (cognitive neuroscience) | - | - | - | - | + | - | - | + |
| iPST | Arean (2016)  Pratap (2018) | CBT | - | - | + | - | - | - | - | + |
| MoodHacker | Birney (2016) | CBT | + | + | + | - | - | - | - | + |
| MCT & More | Bruhns (2021) | CBT and others (MCT and third-wave approaches) | - | - | + | - | + | - | - | + |
| ProACT-S | Chan (2021) | CBT | + | - | - | + | - | - | - | + |
| Moodivate | Dahne (2019a) | BA | + | + | - | - | + | - | - | - |
| MoodKit | Dahne (2019a) | CBT | - | + | - | - | - | - | - | - |
| Aptívate | Dahne (2019b) | BA | - | + | - | - | + | - | - | - |
| iCouch CBT | Dahne (2019b) | CBT | - | + | - | - | - | - | - | - |
| GET.ON Mood | Ebert (2018) | CBT | + | - | + | + | - | - | + | + |
| IntelliCare | Graham (2020) | CBT | + | - | + | - | - | - | + | + |
| Run4Love (WeChat) | Guo (2020) | CBT | - | - | + | + | - | - | + | + |
| HARUToday | Ham (2019) | CBT | + | + | - | + | + | - | - | + |
| Happy Mom | Jannati (2020) | CBT | + | - | + | - | - | - | - | + |
| SPSRS | Kageyama (2021) | Other (Verbal stimulation) | - | - | - | - | - | - | - | + |
| Be Good to Yourself | Lüdtke (2018a) | CBT and others (third-wave approaches) | + | - | - | - | + | - | - | + |
| XiaoNan (WeChat) | Liu (2022) | CBT | + | - | - | - | - | - | - | - |
| MT-Phoenix | Lukas (2021a)  Lukas (2021b) | CBT and others (AAMT) | + | - | - | + | + | - | + | - |
| Unspecified | Ly (2015) | BA | + | - | + | + | + | - | + | + |
| Kokoro | Mantani (2017) | CBT and others (BA) | + | + | + | + | + | - | - | - |
| Meru Health Program | Raevuori (2021) | CBT and others (BA and MBSR) | + | - | - | - | - | + | + | - |
| SuperBetter | Roepke (2015) | Acceptance based | - | - | + | - | + | - | - | + |
| SuperBetter CBT-PPT | Roepke (2015) | CBT | - | - | + | - | + | - | - | + |
| eMums Plus | Sawyer (2019) | CBT and others (attachment theory) | - | - | - | - | - | + | + | - |
| Boost Me | Stiles-Shields (2019) | BA | - | + | - | - | - | - | + | + |
| Thought Challenger | Stiles-Shields (2019) | Other (Cognitive Therapy) | - | - | - | - | - | - | + | + |
| iBobbly | Tighe (2016) | Acceptance based | - | + | - | + | - | - | - | - |
| MONSENSO | Tønning (2021) | CBT | + | + | - | + | - | - | + | + |
| Get Happy Program | Watts (2013) | CBT | + | - | - | - | - | - | + | + |
| Lifestyle Hub | Wong (2021) | Other (Transtheoretical model) | + | - | + | - | + | - | - | + |
|  | | | 17/31 | 10/31 | 13/31 | 10/31 | 12/31 | 2/31 | 12/31 | 20/31 |
|  | | | 54.84% | 32.26% | 41.94% | 32.26% | 38.71% | 6.45% | 38.71% | 64.52% |
| AAMT = Approach-Avoidance Modification Training; BA = Behavioral Activation; CBT = Cognitive Behavioral Therapy; MBSR = Mindfulness Based Stress Reduction; MCT = Metacognitive Therapy; | | | | | | | | | | |
