# Supplementary material for: Efficacy, Safety, and Evaluation Criteria of mHealth Interventions for Depression: Systematic Review
Source: JMIR Ment Health. 2023 Sep 27;10:e46877. doi: 10.2196/46877 (PMC10568392; doi:10.2196/46877)
Supplement: Multimedia Appendix 4 [file mental_v10i1e46877_app4.docx]

Multimedia Appendix 4. Galbraith plot
