# Supplementary material for: Efficacy, Safety, and Evaluation Criteria of mHealth Interventions for Depression: Systematic Review
Source: JMIR Ment Health. 2023 Sep 27;10:e46877. doi: 10.2196/46877 (PMC10568392; doi:10.2196/46877)
Supplement: Multimedia Appendix 5 [file mental_v10i1e46877_app5.docx]

Multimedia Appendix 5. Safety of mHealth interventions

| **Author (year)** | **Measure** | **Results** |
| --- | --- | --- |
| Araya (2021a) | Averse events monitoring | - Brazil: In the intervention group, worsening of depressive symptoms occurred in 13.4% of patients and worsening of suicide ideation occurred in 7.1% of patients vs worsening by 15% and 6.6% in the control group. |
| Araya (2021b) | Averse events monitoring | - Peru: In the intervention group, worsening of depressive symptoms occurred in 2.8% of patients and worsening of suicide ideation occurred in 3.7% of patients vs worsening by 5.1% and 7.4% in the control group. |
| Birney (2016) | Averse events monitoring | - No subjects reported suicide risk severe enough to transfer to a suicide hotline. Nor did any subjects report any adverse events related to the use of the MoodHacker app via email or during follow-up calls. |
| Bruhns (2021) | Inventory for Assessing Negative Effects of Psychotherapy (INEP) | - Overall, of the 119 participants 27 (22.7%) reported a negative side effect. - Fear of stigmatization was the most common negative side effect (12/119, 10.1%). In addition, 6.7% (8/119) of participants reported that they had longer phases in which they felt bad, 5.9% (7/119) of participants reported that they had problems with insurance, 5% (6/119) of participants reported that they experienced more pain from events from the past, 1.7% (2/119) of participants reported that they felt worse, 1.7% (2/119) of participants reported that they were more concerned about financial issues, 0.8% (1/119) of participants reported that trusting others is more difficult for them, 0.8% (1/119) of participants reported that they had a worse relationship with their family, and 0.8% (1/119) of participants reported that they had a worse relationship with their friends. - None of the participants stated that they had changed as a person to the negative, that they had suicidal thoughts or intentions for the first time, or that they experienced more conflicts in their partnership. |
| Chan (2021) | Averse events monitoring | - No adverse events, increased suicidal risk, nor significant deterioration in primary outcome measures was reported throughout the study. |
| Graham (2020) | Averse events monitoring | - There were no study-related adverse events. |
| Guo (2020) | Averse events monitoring | - No adverse events were reported. |
| Kageyama (2021) | Averse events monitoring | - None of the participants discontinued the study due to adverse events after the start of the intervention. |
| Liu (2022) | Averse events monitoring | - No adverse events were observed in the trial. |
| Mantani (2017) | Frequency, Intensity, and Burden of Side Effects Ratings (FIBSER) | - With regard to the harm outcomes, patients using the smartphone CBT reported somewhat less overall burden of side effects, but the difference was not statistically significant (FIBSER mean difference=–0.76, 95% CI –1.58 to 0.05, P=.07). There was one report of suicidality (self-injurious behavior without suicidal intent) in the combined treatment arm and one report of a serious adverse event in the control arm (brief hospital admission for examination of preexisting spinal canal stenosis). |
| Pratap (2018) | Averse events monitoring | - A small proportion (11.48%) deteriorated (PHQ-9 worsened ≥5 points) during the course of the study. However, there was no difference in depression outcomes among the 3 intervention arms. |
| Raevuori (2021) | Averse events monitoring | - There were no study-related adverse events. |
| Stiles-Shields (2019) | Averse events monitoring | - There were no adverse events (e.g., severe suicidality). |
| Wong (2021) | Averse events monitoring | - No adverse event was reported throughout the study. |
