# Supplementary material for: Efficacy, Safety, and Evaluation Criteria of mHealth Interventions for Depression: Systematic Review
Source: JMIR Ment Health. 2023 Sep 27;10:e46877. doi: 10.2196/46877 (PMC10568392; doi:10.2196/46877)
Supplement: Multimedia Appendix 6 [file mental_v10i1e46877_app6.docx]

Multimedia Appendix 6

Table S1. Secondary outcome results from the selected studies

| **Outcome** | **Measure** | **Reference** | **Main result** |
| --- | --- | --- | --- |
| Quality of life (n = 8) | Euroqol Group Quality of Life (EQ-5D-3L) | Araya (2021a) | - At 3 months, there were statistically significant differences between groups in favor of the digital intervention group for quality of life (EQ5D-3L) - At 6 months, there were no statistically significant differences between groups in the EQ-5D-3L |
|  |  | Araya (2021b) | - At 3 months, there were statistically significant differences between groups in favor of the digital intervention group for quality of life (EQ5D-3L) - At 6 months, there were no statistically significant differences between groups in the EQ-5D-3L |
|  | The World Health Organization Quality of Life – Abbreviated Version (WHOQOL-BREF) | Bruhns (2021) | - For both groups (mHealth and waiting list control), the results did not show a significant increase in quality of life (WHOQOL-BREF) - There was no significant improvement across time in quality of life (WHOQOL-BREF). |
|  |  | Guo (2020) | - At the 3-month follow-up, participants in the Run4Love intervention group, when compared with the control group, had significantly improved QOL (WHOQOL-HIV BREF: from 77.4 to 82.6 vs 76.6 to 77.0; mean difference=4.79, 95% CI 2.72 to 6.87; P<0.001) |
|  |  | Lüdtke (2018a) | - The combined model-estimated marginal means of the pre- post change scores and corresponding standard errors (in brackets) are as follows: WHOQOL-BREF: change score = −2.30 (1.43) for the wait-list group vs. change score = −1.57 (1.62) for the intervention group. No significant between-group differences. |
|  |  | Tonning (2021) | - The intervention group had a tendency towards higher quality of life (p=0.07) compared with the control group, |
|  | Quality of Life Inventory (QOLI) | Ly (2015) | - No significant interaction effects for group and time were found on the secondary measures neither between pre-treatment to post-treatment (QOLI: (F1, 88.32 = 0.31, p = .58)) nor between pre-treatment to follow-up treatment (QOLI: (F1, 165.17 = 1.06, p = .31) |
|  | EUROHIS-Qol 8-item index (EUROHIS-Qol-8) | Raevuori (2021) | - No statistically significant differences between the intervention group and the control group in change of quality of life by EUROHIS-Qol-8 were detected. |
|  | Short Form Survey (SF-12) | Chan (2021) | - Post-hoc t tests found no between and within-group differences across the follow-up time points on subjective physical health. |
|  | Short Form Survey (SF-36) | Ham (2019) | - No significant group differences in terms of SF-36 were found (SF-36: F = 2.09, p = 0.132). |
|  |  | Wong (2021) | - The intention-to-treat analysis revealed significant improvements in health-related quality of life (HRQoL; d = 0.11) from Week 0 (baseline) to Week 9 (immediate post- intervention assessment) in the LMG relative to the WLG. |
| Disability | The World Health Organization Disability Assessment Schedule-II (WHODAS-II) | Araya (2021a) | - At 3 months, there were statistically significant differences between groups in favor of the digital intervention group for disability (WHODAS-II) - At 6 months, there were no statistically significant differences between groups in the WHODAS-II |
|  |  | Araya (2021b) | - At 3 months, there were statistically significant differences between groups in favor of the digital intervention group for disability (WHODAS-II) - At 6 months, there were no statistically significant differences between groups in the WHODAS-II |
|  | Disability Symptom Severity (DSS) | Arean (2016) | - Disability decreased an average of 0.67 points per week during weeks 1-4 and did not significantly change from weeks 4-12. The Project: EVO and iPST groups’ disability did not significantly differ from controls at week 4 or week 8 or in the rates of change over time. |
|  | Sheehan Disability Scale (SDS) | Pratap (2018) | - At the cohort level, disability based on SDS ratings decreased by an average 0.74 points (P=.03) in weeks 2-4 and further by 0.39 points (beta=−1.09, P=.02) in weeks 5-12. There was no difference in disability outcomes across treatment arms. |
|  |  | Watts (2013) | - Statistical tests using the SDS showed a significant reduction in the number of days lost (absenteeism) (Wald Chi-Square =10.31, P = <.05) and in the number of days underproductive (presenteeism) from pre-treatment to post-treatment (Wald Chi-Square =12.33, P = .001). - Further results indicated that the interaction between experimental group and time was not statistically significant when comparing the SDS on absenteeism (Wald Chi- Square =.86, P = .35) and presenteeism (Wald Chi-Square =.22, P = .63). |
|  |  | Wong (2021) | - The intention-to-treat analysis revealed significant improvements in functional impairment (d = 0.22), from Week 0 (baseline) to Week 9 (immediate post- intervention assessment) in the LMG relative to the WLG. |
| Behavioral Activation | Behavioral Activation for Depression Scale-Short Form (BADS-SF) | Araya (2021a) | - At 3 months, there were no statistically significant differences between groups in the BADS-SF - At 6 months, there was a statistically significant difference in favor of the digital intervention group in the BADS-SF total score |
|  |  | Araya (2021b) | - At 3 months, there were statistically significant differences between groups in favor of the digital intervention group for behavioral activation (BADS-SF) - At 6 months, there was a statistically significant difference in favor of the digital intervention group in the BADS-SF total score |
|  |  | Ebert (2018) | - There were significant between-group differences for activation favoring the intervention group (d = 0.63, 95%CI 0.35, 0.91). |
|  |  | Toning (2021) | - The intervention group had a tendency towards higher behavioral activation (p=0.08) compared with the control group |
| Negative Thinking | Automatic Thoughts Questionnaire-Revised (ATQ-R) | Birney (2016) | - From pretest to 6-week follow-up, the ANCOVA with the full sample found statistically significant positive effects for the program on ATQ-R (p=0.01, partial eta2 = 0.020) |
| Knowledge | Self-developed scale | Birney (2016) | - From pretest to 6-week follow-up, the ANCOVA with the full sample found statistically significant positive effects for the program on knowledge (p=0.02, partial eta2 = 0.017) |
| Self-esteem | Rosenberg Self-esteem Scale (RSE) | Bruhns (2021) | - Within group differences: Results of paired samples t tests indicated a significant increase in scores on the self-esteem scale (RSE) for the intervention group (p<0.001; d=0.38) and the wait-list control group (p=.001; d=0.16). - Between group differences: The analyses resulted in a medium to large effect size for the increase in self-esteem (d=0.77) across time. |
|  |  | Lüdtke (2018a) | - The combined model-estimated marginal means of the pre- post change scores and corresponding standard errors (in brackets) are as follows. RSE: change score = −2.97 (1.12) for the wait-list group vs. change score = −1.20 (1.11) for the intervention group. - However, no pre-post between-group differences were found (p = 0.738) |
| Anxiety | Hospital Anxiety and Depression Scale (HADS) | Chan (2021) | - Withing group differences: In the within-group comparison, the treatment group showed medium-to-large difference before and after the treatment, p<0.001, d=0.64, while the control group showed no significant change. - Between group differences: Between-group comparison at week 6 follow-up was significant, in favor of the treatment group, p < 0.001, d = 0.83. |
|  |  | Ebert (2018) | - There were significant between-group differences for anxiety favoring the intervention group (d = 0.71, 95%CI 0.43, 0.99). |
|  | State-Trait Anxiety Inventory (STAI) | Ham (2019) | - Significant differences were also found across groups in state anxiety (F = 10.44, p = 0.001); hence, a post hoc test was conducted that revealed a significant decrease in state anxiety in the intervention group and attention control group compared to the waitlist control group (95% CI [5.81, 19.51], p = 0.000, ηp2 = 0.59; 95% CI [0.38, 14.08], p = 0.035, ηp2 = 1.32). - There was significant differences across the groups before and after the intervention in trait anxiety (F = 3.98, p = 0.024); hence, a post hoc test was conducted that revealed a significant decrease in trait anxiety in the intervention group compared to the waitlist control group (95% CI [0.78, 11.91], p = 0.20, ηp 2 = 0.88). |
|  | Generalized Anxiety Disorder-7 (GAD-7) | Kageyama (2021) | - The time (P = 0.12), group (P = 0.28), and group × time interactions (P = 0.25) for the GAD-7 scores were non-significant. |
|  |  | Liu (2022) | - Univariate ANCOVA revealed that chatbot-enhanced self-help intervention showed a significant advantage to bibliotherapy in terms of the reduction of anxiety with a low effect size (d = 0.30) as measured by the GAD-7 (F = 5.37; P = 0.02) in the 16-week period. |
|  |  | Raevuori (2021) | - No statistically significant differences between the intervention group and the control group in change of anxiety by GAD-7, sleep disturbances by ISI, and quality of life by EUROHIS-Qol-8 were detected. |
|  |  | Roepke (2015) | - Of note is that SB users experienced greater decreases in anxiety than WL |
|  |  | Wong (2021) | - The intention-to-treat analysis revealed significant improvements in generalized anxiety symptoms (d = 0.93), from Week 0 (baseline) to Week 9 (immediate post- intervention assessment) in the LMG relative to the WLG. |
|  | Beck Anxiety Inventory (BAI) | Ly (2015) | - No significant interaction effects for group and time were found on the secondary measures neither between pre-treatment to post-treatment (BAI: (F1, 88.80 = 0.24, p = .62); nor between pre-treatment to follow-up treatment (BAI: (F1, 162.05 = 0.34, p = .56); |
| Problem solving | Social Problem-Solving Inventory-Revised (SPSI-R) | Ebert (2018) | - There were not significant between-group differences. |
| Mastery | Pearlin Mastery Scale (PSMS) | Ebert (2018) | - There were not significant between-group differences. |
| Insomnia | Insomnia Severity Index (ISI) | Chan (2021) | - Within-group differences: the treatment group showed a large difference before and after the treatment, p < 0.001, Cohen’s d = 0.93, 95% Cl (2.42–3.85). The change in the control group corresponded to a small effect size, p = 0.018, Cohen’s d = 0.22, 95% Cl (0.10–1.04) - Between-group differences at week 6 follow-up was significant, in favor of the treatment group (Cohen’s d = 0.91, 95% Cl (−3.34 to −1.83)). |
|  |  | Ebert (2018) | - There were significant between-group differences for insomnia favoring the intervention group (d = 0.41, 95%CI 0.14, 0.69). |
|  |  | Raevuori (2021) | - No statistically significant differences between the intervention group and the control group in change of sleep disturbances by ISI were detected. |
|  |  | Wong (2021) | - The intention-to-treat analysis revealed significant improvements in insomnia symptoms (d = 0.20), from Week 0 (baseline) to Week 9 (immediate post- intervention assessment) in the LMG relative to the WLG. |
| Self-efficacy | General Self-Efficacy Scale (GSES) | Guo (2020) | - At the 3-month follow-up, participants in the Run4Love intervention group, when compared with the control group, had significantly improved self-efficacy (GSES: from 24.4 to 26.6 vs from 23.3 to 23.4; mean difference=2.16, 95% CI 0.92 to 3.40; P<0.001) |
|  | Self-Efficacy Scale (NGSE) | Roepke (2015) | - SB users experienced greater improvements in self-efficacy, than WL. |
|  | Parenting Sense of Competence Scale (PSCS) | Sawyer (2019) | - There were no significant differences in the intervention and standard care groups in scores on the PSCS (P=.11). |
| Perceived stress and distress | Perceived Stress Scale (PSS) | Guo (2020) | - In comparison with the control group, participants in the intervention group also had significantly reduced perceived stress (PSS: from 20.0 to 15.7 vs from 20.7 to 18.9; mean difference=−2.45, 95% CI −3.63 to −1.27; P<.001) |
|  |  | Raevuori (2021) | - The reduction of the perceived stress by PSS-10 was larger in the intervention group than in the control group at T20 and at T32 (LSM difference -2.71, 95% CI = -4.73, -0.70, p = 0.008, Cohen’s d = -0.52). |
|  |  | Tonning (2021) | - No significant effect. |
|  | Parenting Stress Index (PSI) | Sawyer (2019) | - There were no significant differences in the intervention and standard care groups in scores on the PSI competence subscale (P=.69) |
|  | Kessler Screening Scale for Psychological Distress (K-6) | Kageyama (2021) | - The effect of time on the secondary outcome (K-6 score) was significant (P = 0.01), whereas the group (P = 0.82) and group × time interactions (P = 0.37) were non-significant. |
|  | Kessler Screening Scale for Psychological Distress (K-10) | Tighe (2016) | - Participants in the ibobbly arm also showed a substantial and statistically significant reduction in K10 scores compared with waitlist controls. The interaction of intervention arm by time (preintervention vs postintervention) was significant (t=2.44; df=57.5; p=0.0177). Cohen’s d was 0.65 (95% CI 0.12 to 1.17), reflecting a substantial effect. |
|  |  | Watts (2013) | - Statistical tests showed that the benefits of the intervention remained significant in both groups at follow up when using the K-10 (F [7, 1734.5] = 28.4, P=<.001). |
| Coping | Simplified Ways of Coping Questionnaire (SWCQ) | Guo (2020) | - At the 3-month follow-up, participants in the Run4Love intervention group, when compared with the control group, had significantly improved SWCQ positive coping (from 18.4 to 20.7 vs from 18.3 to 17.8; mean difference=2.91, 95% CI 1.39 to 4.43; P<.001) - There were no significant between-group differences in changes in SWCQ negative coping. |
| Physical activity | Global Physical Activity Questionnaire (GPAQ) | Guo (2020) | - There were no significant between-group differences in changes in physical activity (METs; P>.005). |
| Dysfunctional Attitudes | Dysfunctional Attitudes Scale (DAS) | Ham (2019) | - No significant group differences in terms of DAS (F = 0.16, p = 0.849). |
| Affect | The Positive and Negative Affect Schedule (PANAS) | Liu (2022) | - No significant between-group difference was observed on positive and negative affect. |
| Wellbeing | World Health Organization Well-being Index (WHO-5) | Lukas (2021b) | - With regard to well-being, ANCOVA results showed significant differences between the intervention and the waitlist control condition at postintervention assessment (F1,74=15.34; P=.001) with a large effect (d=0.91). |
|  |  | Tonning (2021) | - The intervention group had tendency towards higher well- being (p=0,09) compared with the control group |
| Psychological inflexibility and experiential avoidance | Acceptance and Action Questionnaire (AAQ-II) | Ly (2015) | - No significant interaction effects for group and time were found on the secondary measures neither between pre-treatment to post-treatment (AAQ-II: (F1, 90.20 = 0.16, p = .70) nor between pre-treatment to follow-up treatment (AAQ-II: (F1, 166.88 = 0.01, p = .91) |
| Resilience | Resilience Scale | Raevuori (2021) | - The increase in Resilience Scale was larger in the intervention group than in the control group at T32 (LSM difference 3.63, 95% CI = 0.44, 6.82, p = 0.03, Cohen’s d = 0.32) |
| Satisfaction with Life | Satisfaction with Life  Scale | Roepke (2015) | - SB users experienced greater improvements in life satisfaction, than WL. |
| Impulsivity | The Barratt Impulsivity Scale (BIS-11) | Tighe (2016) | - Preintervention scores on the BIS-11 for the waitlist group were significantly lower than for the ibobbly group (t=2.05; df=59.2; p=0.0446). Postintervention means were identical. Scores in the waitlist decreased from postintervention to follow-up although this was not significant (t=−1.82; df=29.1; p=0.0792). |
| Suicidal ideation | The Depressive Symptom Inventory—Suicidality Subscale (DSI-SS) | Tighe (2016) | - Although preintervention and postintervention changes were significant in the ibobbly arm (t=2.40; df=58.1; p=0.0195), the interaction of intervention arm by time (Preintervention vs postintervention) was not significant (t=1.05; df=57.8; p=0.2962). |
| Psychological functioning | Psychosocial functioning according to the Functional Assessment Short Test (FAST) | Tonning (2021) | - No significant effect. |
| Empowerment | Roger’s Empowerment Scale | Tonning (2021) | - No significant effect. |
| Worry | Penn State Worry Questionnaire (PSWQ) | Toning (2021) | - No significant effect. |

Table S2. Output tools and results from the selected studies

| **Output** | **Measure** | **Reference** | **Main result** |
| --- | --- | --- | --- |
| Intervention adherence and app utilization | - | Arean (2016) | - Among the 420 participants in the Project: EVO and iPST conditions, 243 (57.9%) did not download their assign app. On average, those who used their app at least once used it 10.78 ±11.44 times. |
|  | - | Bruhns (2021) | - The self-help smartphone app was used by 23.3% (28/400) of participants daily, by 17.5% (21/400) of participants 4-6 times a week, by 25% (30/400) of participants 2-3 times a week, by 10% (12/400) of participants once a week, by 19.2% (23/400) of participants 1-3 times in total, and by 5% (6/400) of participants not at all. - The improvement in symptoms (PHQ-9) did not correlate with use frequency (r=0.020; p=0.83). |
|  | - | Birney (2016) | - On average, participants in the treatment arm logged into the MoodHacker app 16.0 times ± 13.3 (range 1-49) for a total duration of 1.3 hours ±1.3 (range 0-6.5) between pretest and 6-week follow-up. |
|  | - | Dahne (2019a) | - All participants used the app at least once during the trial, 71.4% of participants used the app at least 28 times, and 42.9% of participants used the app more than 56 times. - Participants on average had 46.76 ± 30.10 app sessions throughout the eight-week trial duration, spent 3.50 ± 2.76 minutes using the app per session, and spent 120.76 ±101.02 minutes using the app in total throughout the trial. - Participants created on average 6.10 ± 3.22 unique values within the app, 14.71 ± 10.22 activities across values, and completed 52.24 ± 89.31 activities - In general, retention was high across the study duration: 88.9% of participants utilized Moodivate at least once during the first week following trial enrollment, 83.3% during the second week, 66.7% during the third week, 66.7% during the fourth week, 66.7% during the fifth week, 66.7% during the sixth week, 61.1% during the seventh week, and 50.0% during the eighth week. |
|  | - | Dahne (2019b) | - Aptívate! participants used the app at least once during the trial, 81.8% of participants used the app at least 8 times, 45.5% of participants used the app at least 28 times (i.e., every other day on average), and 36.4% of participants used the app 56 or more times. - Participants on average had 61.41 ± 91.73 app sessions throughout the eight-week trial duration, spent 87.73 ± 63.08 seconds using the app per session, and spent 65.77 ± 82.76 minutes using the app in total throughout the trial. - Participants created on average 4.68 ± 3.54 unique values within the app, 11.77 ± 17.02 activities across values, and completed 21.73 ± 45.60 activities - In general, retention was high across the study duration: 100% of participants utilized ¡Aptívate! at least once during the first week following trial enrollment, 77.3% during the second week, 77.3% during the third week, 72.7% during the fourth week, 59.1% during the fifth week, 54.5% during the sixth week, 59.1% during the seventh week, and 50.0% during the eighth week. |
|  | - | Ebert (2018) | - The average treatment duration was 7 weeks (SD = 3.17) and participants completed on average 5 sessions (SD = 2.25). Out of the 102 participants who were initially assigned to the intervention, 68 (66.7%) were intervention completers. Of those, 63 (92.6%) adhered to all six sessions. - The booster session was completed by 40 (39.2%) participants. Of the 34 participants (33.3%) not completing 80% of the intervention, 6 participants never started the intervention (5.9%). |
|  | - | Graham (2020) | - Neither app sessions (r, −0.03; 95% CI, −0.22 to 0.15), time to last use (r, −0.14; 95% CI, −0.31 to 0.05), nor days used (r, −0.05; 95% CI, −0.23 to 0.14) were strongly associated with changes in depression. - At 8 weeks following treatment, 119 participants (81.5%) had some app use. For all participants, postintervention median time to last app use was 28 days (range, 0-212 days) and median days used was 7 (range, 0-102 days). |
|  | - | Kageyama (2021) | - The mean and SD of video viewing time during the 5-week intervention was 356.59 ± 351.55 min. Adherence rate was calculated by dividing the number of participants who achieved the required viewing time for the duration of the intervention by the number of participants in the experimental group. Of the 16 participants, five complied with the application intervention. Thus, an adherence rate of 31% was achieved. |
|  | - | Liu (2022) | - The self-reported adherence rates of the chatbot test group and bibliotherapy group were 1.96 (SD = 0.70) and 1.97(0.62), respectively. Independent t-test detected no significant difference between the adherence rates of the two groups (t = 0.68; P = 0.50). |
|  | - | Lüdtke (2018a) | - Despite the high completion rate, only 39% of the participants in the intervention group actually used the application frequently (i.e., several times a week). Willingness to change was not correlated with self-reported frequency of usage (r = 0.05, p = 0.805). |
|  | - | Lukas (2021a) | - Participants used the app on average for 8.2 days (SD = 3.56, range = 3–11) for an average of 41.31 min (SD = 39.86, range = 8.8–121.87). Participants completed an average of 9.4 training sessions (SD = 4.28, range = 3–13). |
|  | - | Lukas (2021b) | - With regard to intervention engagement, 13 participants did not initiate training with the intervention over the intervention period. The 27 participants that did initiate training with MT-Phoenix used the app for an average of 6.38 days (SD 2.83) and spent 62.39 minutes in the app (SD 68.17). During the training, participants completed 5.89 (SD 4.43) modules and an average of 25.89 (SD 23.43) tasks and played 2.28 (SD 3.10) levels of the AAMT per module. |
|  | - | Ly (2015) | - Adherence to treatment was defined as the number of completed face-to-face sessions. Out of 88 participants, who started the treatment, 81 (92%) succeeded to adhere to the entire treatment. 42 (93.3%) participants of those were in the blended treatment group and 39 (92.9%) were in the full BA treatment group. |
|  | - | Mantani (2017) | - For the smartphone CBT, all but one participant (80/81, 99%) completed at least half the sessions of the program, and 71 of 81 (88%) completed at least six of eight sessions. It took the patients, on average, 10.8 (SD 4.2) days to complete one session. |
|  | - | Roepke (2015) | - Treatment adherence was lower than might be expected in traditional clinical randomized controlled trials, likely because of the absence of incentives. Of 190 participants assigned to use SB, 75.80 percent (n = 144) logged in at least once. Number of log-ins ranged from 1 to 274 total, with a mean of 21.53 (SD = 34.27) and median of 9.50. |
|  | - | Sawwyer (2019) | - In the first 11 weeks of the intervention, more than 60% (43/72) of participants logged into the intervention at least once each week. - more than 50% (38/72) of mothers logged into the intervention at least once each week until the 14th week of the 16-week intervention. |
|  | - | Stiles-Shields (2019) | - Boost Me was launched significantly more than Thought Challenger (97.7 vs. 33.5, t(18) = 2.59, p = .02, d = 1.16). No significant differences emerged in the number of events (Boost Me; 14.7 ± 10.1) and thoughts (Thought Challenger; 8.5 ± 11.6) logged overall (p = .22, d = .57), nor in event (7.5 ± 7.3) or thought (5.4 ± 4.5) reviews (p = .45, d = .35). - App usage was not significantly correlated with changes in depression scores |
|  | - | Tighe (2016) | - Of the 40 participants for whom usage data were available, 34 (85%) completed all activities (three self-assessments and three content modules), 1 completed five out of six activities (three self-assessments and two content modules), and 5 completed two out of six activities (one self-assessment and one content module). This shows good adherence to the trial by those whose usage data were available |
|  | - | Watts (2013) | - 8.6% (3/35) completed only the first lesson, 2.9% (1/35) completed two lessons, 2.9% (1/35) completed 3 lessons, 5.7% (2/35) completed 4 lessons, 11.4% (4/35) completed 5 lessons and 68.6% (24/35) of participants completed all six lessons. When comparing the Mobile Group with the Computer Group on adherence, there were no significant differences (t (33) = −.242, P >.05). |
| Satisfaction | Likert scale | Birney (2016) | - The average rating of program satisfaction was 4.6 ± 1.0 on a 6-point scale, indicating that the participants were mostly satisfied with the intervention. |
|  | Client Satisfaction Questionnaire-8 | Bruhns (2021) | - The average total score was mean 20.28 ± 5.36; (8=very dissatisfied to 32=very satisfied). The quality of the self-help smartphone app was rated positively by 64.7% (77/119) of participants. For each item, the positive evaluations outweighed the negative evaluations. |
|  |  | Lüdtke (2018a) | - The results of the Client Satisfaction Questionnaire (ZUF-8) suggest that users evaluated the application positively. |
|  | System Usability Scale (SUS) | Lukas (2021a) | - Regarding the evaluation of the acceptance and usability of MT-Phoenix, participants reported a high degree of satisfaction (M = 91.25, SD = 2.06) on the SUS. |
|  | Multi-choice questionnaire | Ham (2019) | - Results showed that the overall satisfaction of the intervention group (HARUToday) was significantly higher than that of the attention control group (U = 123.50, p = 0.029). |
|  | the Verona Satisfaction Scale-Affective Disorder (VSS-A) | Toning (2021) | - The intervention group had a tendency towards higher satisfaction with treatment (p=0.05) compared with the control group |
|  | Credibility/Expectancy Questionnaire (CEQ) | Watts (2013) | - Upon completion of the program 54% of Mobile Group and 64% of the Computer Group were very satisfied with the program; with the remaining participants endorsing ‘somewhat satisfied’. 64% of the Mobile Group and 64% of the Computer Group would be very confident in recommending this treatment to a friend; and the remainder endorsed ‘somewhat confident’. |
| Acceptability and Usability | System Usability Scale (SUS) | Birney (2016) | - The average SUS score was 79.7 ± 17.1, corresponding to a usability grade of B+ for the intervention program. |
|  |  | Raevuori (2021) | - The mean SUS score of the MHP was 86.9 (median 87.5, range 60.0- 100.0), suggesting above average usability (cut-off 68 points) among respondents (n = 44, 69.8% of the intervention group). |
|  |  | Stiles-Shields (2019) | - Mid-treatment (week 3) mean SUS scores indicated that Thought Challenger (84.10 - ± 10.43) was rated significantly higher than Boost Me (70.00 ± 14.31; t(15) = −2.29, p = .04, d = 1.12). However, at end of treatment (week 6), there was no significant difference in mean SUS scores between Thought Challenger (88.57 ± 5.56) and Boost Me (78.33 ± 15.10; t(14) = −1.70, p = .11, d = .90). |
|  | Participant Acceptability / Usability Rating Scale | Chan (2021) | - Over half of the participants found proACT-S easy to use. Nearly 90% of the participants found proACT-S clear and easy to understand. - Over 60% of the participants were satisfied with the experience using proACT-S and nearly 70% would like to continue to use proACT-S if it was available. Half of the participants found it helpful in managing their symptoms. |
|  | Self-developed questionnaire | Sawyer (2019) | - With the exception of mood graphing and video components of the app, the majority of mothers who used each component also reported that it was very easy or easy to use. However, a large percentage (30% (18/60) to 47% (28/60)) of mothers did not use some key components of the app such as the mood-rater designed to allow mothers to track their mood level over time and activities embedded in topic areas that were designed to help improve maternal emotional health. Finally, 90% (52/58) of mothers reported that the length of the information in each topic area was about right. In addition, 44% (25/57) of mothers reported the length of the intervention was about right, whereas 51% (29/57) reported that it was too short. |
| Attitude Toward Intervention | Attitude Toward Psychological Online Interventions (APOI) | Bruhns (2021) | - In total, of the 400 participants, 232 (58%) had a positive attitude toward internet- and mobile-based interventions, 30 (7.5%) had a neutral attitude toward internet- and mobile-based interventions, and 138 (34.5%) had a negative attitude toward internet- and mobile-based interventions. |
| Therapy Expectations | The Patient Questionnaire on Therapy Expectation and Evaluation (PATHEV) | Bruhns (2021) | - Of the 400 participants, 257 (64.3%) indicated a positive expectation and 117 (29.3%) indicated a negative expectation of treatment outcome regarding the self-help smartphone app. Approximately half of the participants (191/400, 47.8%) did not expect the self-help smartphone app to reduce their symptoms and indicated that this is not the right program for them (172/400, 43%). - The effectiveness of the app could not be predicted by attitudes. |
|  | Credibility-  Expectancy Questionnaire (CEQ) | Wong (2021) | - The paired samples t-test revealed no significant difference in intervention credibility, t(38) = –1.72, p = .09, and intervention expectancy, t(38) = –0.90, p = .37, from baseline to Week 9 in the LMG. |
| Usefulness | Likert scale | Lukas (2021b) | - Evaluation results were above average, with high ratings for psychoeducation (mean 3.17, SD 0.72), the approach-avoidance bias modification training (mean 3.00, SD 0.53), and behavioral activation tasks (mean 3.17, SD 0.55). |
|  | Self-developed questionnaire | Saywer (2019) | - In all areas, the majority of mothers reported that intervention components were very helpful or helpful. |
| Alliance | Working Alliance Inventory (WAI) | Ly (2015) | - The results of the Working Alliance Inventory showed no significant differences between both groups (t91 = −0.32 to t77 = 0.89, p = .75 to .37). |
| Credibility | C-scale | Ly (2015) | - Treatment credibility ratings (C-scale) after one week of treatment showed that participants in both groups rated their respective treatment as credible. Out of a possible total of 50, the aver- age scores were 33.0 (SD = 8.0) for the blended treatment group and 33.5 (SD = 6.5) for the full BA group. Independent t-tests showed no significant differences between both groups at the C- scale (t84 = −0.30, p = 0.76) |
|  | Credibility-  Expectancy Questionnaire (CEQ) | Wong (2021) | - The paired samples t-test revealed no significant difference in intervention credibility, t(38) = –1.72, p = .09, and intervention expectancy, t(38) = –0.90, p = .37, from baseline to Week 9 in the LMG. |
